# Allometric equations for orchard and vineyard trees: enhancing AFOLU-based climate change mitigation

**DOI:** 10.3389/fpls.2025.1663283

**Published:** 2025-12-17

**Authors:** Myeongja Kwak, Jongkyu Lee, Hyocheng Cheng, Iereh Kim, Juyeong Kim, Suyoung Woo

**Affiliations:** Department of Environmental Horticulture, University of Seoul, Seoul, Republic of Korea

**Keywords:** allometric equations, aboveground biomass, carbon sequestration, climate mitigation strategies, perennial fruit trees, power function, orchards

## Abstract

**Introduction:**

Perennial orchard systems are emerging as important yet underrepresented carbon sinks within the AFOLU sector, which contributes 20–24% of global GHG emissions. Many countries still rely on Tier 1 default values that fail to capture the structural and management characteristics of orchard species. Accurate biomass and carbon estimation, particularly through species-specific allometric equations, is essential for improving Tier 2–3 GHG reporting and recognizing orchards as meaningful contributors to climate-smart land management.

**Methods:**

A systematic literature review was conducted using five major databases (2008–2024), following PRISMA guidelines. From 240 initial records, 53 studies met the inclusion criteria. These were categorized into three domains: (i) biometric modeling of fruit-tree biomass, (ii) species-specific allometric equation development, and (iii) carbon-sequestration assessments. Methodological trends, model performance, and research gaps were synthesized to inform an IPCC-aligned framework for orchard-specific emission and removal factors.

**Results:**

Most studies were concentrated in Asia and the Mediterranean and focused on citrus, mango, apple, grape, and olive systems. Power-law allometric models dominated and generally showed high predictive performance (R² > 0.90) with variables such as diameter, height, and crown dimensions. However, major gaps remained: limited data for belowground biomass, juvenile trees, grafted architectures, vineyards, and uncertainty quantification—all of which restrict Tier 2–3 applicability.

**Discussion:**

Based on these findings, this review proposes a standardized methodological framework linking biometric measurements, species-specific allometric modeling, remote-sensing integration, and uncertainty analysis to derive orchard-specific emission and removal factors consistent with IPCC guidance. Broader adoption of such protocols would improve transparency and accuracy in national AFOLU inventories and strengthen recognition of perennial orchards as viable nature-based climate solutions that support national net-zero targets.

## 1 Introduction

### 1.1 The role of the AFOLU sector in climate change mitigation

Climate change, driven by the increasing concentration of greenhouse gases (GHG) in the atmosphere, remains one of the most pressing global environmental challenges. Among the sectors contributing significantly to anthropogenic GHG emissions, the Agriculture, Forestry, and Other Land Use (AFOLU) sector sector is particularly important. It is responsible for approximately 20–24% of global GHG emissions, primarily through deforestation, land conversion, and unsustainable agricultural practices (IPCC, 2019). At the same time, the AFOLU holds substantial potential for climate change mitigation through the enhancement of terrestrial carbon sinks—particularly in biomass and soils (de Coninck et al., 2018; Keith et al., 2009; Lee and Lee, 2022).

Beyond forest ecosystems, the contribution of trees outside forests (TOF) and urban green infrastructure is increasingly recognized as a critical component of terrestrial carbon storage. The TOF systems, including roadside trees, windbreaks, home gardens, and scattered trees on agricultural land, play a vital role in capturing atmospheric CO_2_ and maintaining landscape−level carbon balance (Nandal et al., 2023a; Nandal et al., 2023b; Nandal et al., 2024). Similarly, urban soils and vegetation serve as localized carbon sinks, where improved soil structure, organic−matter accumulation, and vegetation management can significantly enhance long−term carbon sequestration potential in cities and peri−urban landscapes.

While much of the mitigation focus within the AFOLU has historically centered on forest ecosystems, perennial orchard systems have emerged as an important but underrepresented component of the global carbon balance. As long-lived agroecosystems, fruit orchards accumulate and store carbon over multiple decades in aboveground woody biomass, root systems, and increasingly, soil organic carbon. Unlike annual crops, these perennial systems contribute to carbon sequestration continuously throughout their lifespan. Empirical studies in regions such as China and the Mediterranean have demonstrated that mature orchards—including apple, citrus, and olive—can function as substantial carbon sinks, often rivaling young or secondary forests in sequestration potential (Scandellari et al., 2016; Wu et al., 2012; Yang et al., 2020; Zanotelli et al., 2015; Zhang et al., 2025).

### 1.2 Challenges in carbon accounting for orchards

The AFOLU sector plays a pivotal role in both contributing to and mitigating anthropogenic GHG emissions. Within this broader AFOLU context, orchard systems remain poorly integrated into national GHG inventory frameworks. The Intergovernmental Panel on Climate Change (IPCC) Guidelines for National Greenhouse Gas Inventories (IPCC, 2006, 2019) offer structured methodologies for estimating emissions and removals, with Tier 2 and Tier 3 levels requiring country-specific or species-specific data and models. However, many countries continue to rely on Tier 1 default values—which are typically derived from forest species or generalized land-use categories—and do not adequately reflect the unique structural, physiological, and management characteristics of fruit orchards.

Estimating biomass and carbon stocks in orchard systems is particularly challenging due to a range of factors. Among these, distinct management practices—such as seasonal pruning and canopy training—can significantly influence aboveground biomass allocation and alter canopy architecture, thereby complicating the applicability of standard estimation models (Barbault et al., 2024; Brym and Ernest, 2018). Species-specific growth patterns and grafted tree architectures require tailored modeling approaches (Quiñones et al., 2013). Differences in planting density and tree age heterogeneity within orchard systems are major factors that complicate the accurate extrapolation of individual tree biomass or carbon stock estimates to area-based units (e.g., per hectare) (Laužikė et al., 2020). Consequently, direct application of forest-derived allometric models to orchard systems can result in substantial bias, undermining the accuracy and transparency of national reporting and mitigation planning.

### 1.3 Importance of greenhouse gas inventories in the LULUCF sector

Among the five major sectors defined by the IPCC—energy, industrial processes, agriculture, waste, and LULUCF (Land Use, Land-Use Change, and Forestry)—the LULUCF sector uniquely functions as both a source and a sink of greenhouse gases. Its inclusion in national GHG inventories is vital for reflecting the mitigation potential of forests, croplands, wetlands, and increasingly, perennial orchard systems such as fruit trees and vineyards. Several countries have recently advanced their inventory approaches for perennial cropping systems, applying differentiated Tier methodologies to enhance transparency and accuracy in carbon accounting (UNFCCC, 2024): Switzerland reported carbon stock changes in low-stem orchards and vineyards using Tier 2 methodologies with country-specific emission and removal factors, improving the resolution of land-use subcategories in the inventory. Germany relied on Tier 1 default values for orchards including apple, cherry, and plum trees, without deploying species-specific models. Australia applied Tier 2 and Tier 3 methods under its National Carbon Accounting System (NCAS) to assess carbon changes in perennial crops such as apples, oranges, almonds, and macadamias, incorporating remote sensing and allometric modeling. Netherlands combined Tier 1 and Tier 2 approaches for orchards, vineyards, and nurseries under the Cropland category, emphasizing uncertainty reduction through stratified land-use classification. These examples reflect a broader international trend toward improving transparency, accuracy, and completeness in LULUCF reporting. As orchard systems gain recognition for their potential role in carbon sequestration, there is growing need to integrate species-specific data, allometric equations, and advanced remote sensing tools to support Tier 2 and Tier 3 inventory development. Given the increasing emphasis on Tier 2 and Tier 3 inventory development under global climate frameworks, there is an urgent need for accurate, orchard-based methods to estimate carbon stock changes.

### 1.4 The role of allometric modeling and the need for a dedicated review

To address these limitations, recent years have seen a growing body of research focused on the development of species-specific allometric equations for fruit trees. These models establish empirical relationships between easily measured biometric variables (e.g., diameter at breast height, tree height, canopy volume) and aboveground biomass. Importantly, they enable non-destructive estimation of carbon stocks at both the individual tree and orchard scales. Studies in citrus (Quiñones et al., 2013; Sahoo et al., 2021), apple (Wu et al., 2012; Zanotelli et al., 2013), mango (Ganeshamurthy et al., 2016; Rupa et al., 2020), and even vineyards (Zhang et al., 2021) have illustrated the feasibility and importance of such models, especially under the growing demand for Tier 2 reporting accuracy. However, the existing literature remains fragmented, with many models developed in isolation, applied only to specific cultivars, or lacking full validation and uncertainty estimates. There is also limited synthesis of how different modeling approaches perform across species, regions, and management regimes, creating barriers to broader policy adoption and standardization. Given these methodological challenges, this review aims to synthesize recent progress in orchard allometry.

### 1.5 Objectives and contributions

This review responds to the urgent need for a structured synthesis of biomass estimation and carbon accounting methodologies tailored to perennial orchard systems. It aims to: (1) Provide a comprehensive overview of published allometric equations for major fruit species, including methodological diversity and performance metrics; (2). Present a transparent, IPCC-aligned framework for developing orchard-specific biomass-based emission and removal factors, emphasizing model validation and uncertainty quantification; (3) Offer representative case studies to highlight species- and system-specific insights in carbon modeling; and (4) Support countries in transitioning from default to Tier 2 reporting by demonstrating feasible, empirically grounded approaches for orchard carbon accounting.

By consolidating and critically analyzing the current state of knowledge, this review contributes to the scientific foundation necessary for integrating perennial orchard systems into national GHG inventories. It also aims to facilitate international harmonization efforts, helping to elevate the role of orchard agroecosystems in global climate change mitigation strategies under the AFOLU framework.

## 2 Materials and methods

### 2.1 Research objectives and questions

This review evaluates species-specific allometric models for perennial fruit trees to improve the accuracy and reliability of biomass and carbon stock estimation in orchard systems. The primary goal is to pinpoint key predictor variables and methodological best practices that can enhance modeling reliability—information that is essential for strengthening GHG inventories and national-level carbon accounting efforts.

To structure the review, the following research questions were formulated: (1) What are the most commonly used variables for estimating biomass in perennial fruit trees? (2) How does the predictive accuracy of existing allometric equations for forest trees compare with those tailored for fruit trees? (3) What methodological approaches are most effective for developing and validating species-specific allometric equations for orchard systems?

### 2.2 Literature search strategy

A systematic literature search was conducted to identify peer−reviewed studies aligned with the review’s scope, specifically focusing on biomass estimation, allometric modeling, and carbon sequestration in perennial fruit−tree systems. Databases included Google Scholar, Web of Science, Scopus, Wiley Online Library, and ScienceDirect, selected for their comprehensive coverage of environmental science, forestry, and agricultural literature. The search covered the period from January 1, 2008, to December 31, 2024, and was restricted to English-language publications only (see **Table 1** for full query details). Search strings combined key terms such as “allometric equations” AND “fruit trees”, “biomass estimation” AND “orchards”, and “carbon sequestration” AND (“vineyards” OR “perennial fruit crops”), using Boolean operators (“AND”, “OR”) and, where applicable, truncation to capture word variants. Screening and data−extraction tasks were conducted independently by three team members (the lead author and two co−authors), and any discrepancies were resolved through joint discussion and consensus.

**Table 1:** Overview of the literature search strategy.

Component	Details
Databases Searched	Google Scholar, Web of Science, Scopus, Wiley Online Library, ScienceDirect
Keywords Used	“allometric equations,” “fruit trees,” “biomass estimation,” “carbon sequestration,” “orchards”
Search Logic	Boolean operators “AND” and “OR” used to refine keyword combinations
Publication Years	2008 to 2024
Document Types Included	Peer-reviewed journal articles, conference proceedings, technical reports

**Figure 1:**
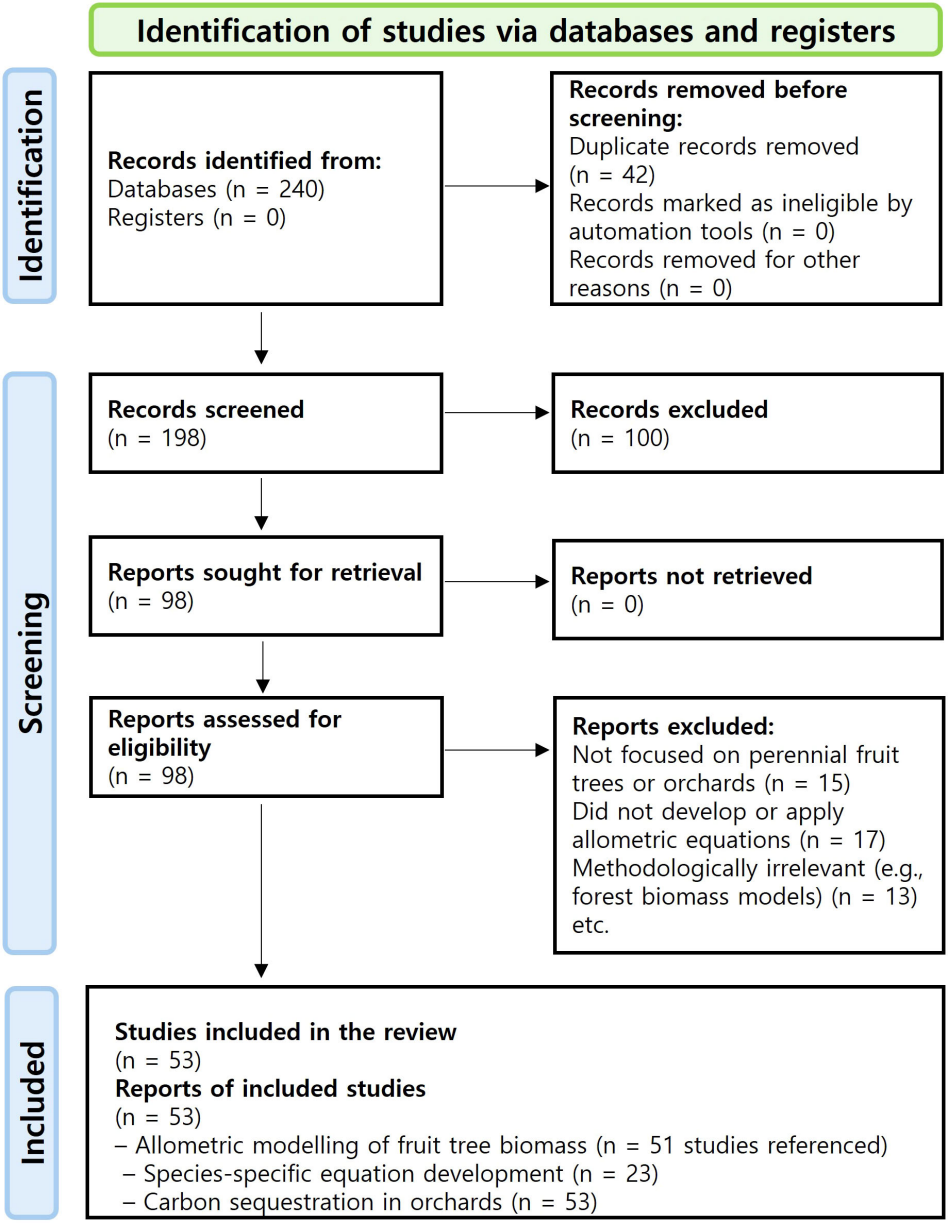
PRISMA-style flow diagram showing identification, screening, eligibility, and inclusion with reasons for exclusions. This diagram illustrates the study selection process conducted in accordance with the PRISMA (Preferred Reporting Items for Systematic Reviews and Meta-Analyses) 2020 guidelines. A total of 240 records were identified from electronic databases. After removing 42 duplicates, 198 unique records were screened for relevance. Of these, 100 records were excluded during the initial screening due to irrelevance or insufficient information. Full-text retrieval was attempted for the remaining 98 records, all of which were successfully assessed for eligibility (n = 53, including additional sources cited within retrieved reports).

Although this review primarily targeted peer−reviewed journal articles, it is important to note that SCI−indexed papers on perennial fruit−tree systems remain relatively limited compared to forestry studies. Therefore, relevant high−quality conference proceedings and technical reports were also incorporated to ensure comprehensive coverage of available research. Studies focusing exclusively on forest trees or annual crops were excluded. The main exclusion criteria at the full−text stage included: not focused on perennial fruit trees or orchards, not developing or applying allometric equations, and methodologically irrelevant studies (e.g., forest biomass models). A PRISMA 2020−style flow diagram (**Figure 1**) illustrates the number of records identified, duplicates removed, records screened, full−text articles assessed for eligibility, excluded studies (with reasons), and final studies included in the review.

### 2.3 Screening and selection criteria

A three-stage screening process was applied to ensure that only studies directly relevant to perennial fruit-tree biomass modeling were included. In the identification stage, titles and abstracts were reviewed to determine topical relevance. During the screening stage, full-text articles that explicitly developed or applied allometric equations to perennial fruit-tree crops were retained. Finally, in the eligibility stage, studies focusing solely on forest ecosystems or annual crops were excluded. From an initial pool of approximately 240 records, 53 studies met all inclusion criteria and were selected for review. These studies were subsequently classified into three thematic domains: (i) biometric modeling of fruit-tree biomass, (ii) development of species-specific allometric equations, and (iii) carbon-sequestration potential in orchard systems.

### 2.4 Methods for estimating carbon stock changes

The IPCC provides a hierarchical approach for estimating GHG emissions and removals in the AFOLU sector. The IPCC Guidelines outline Tier 1 to Tier 3 methodologies, with increasing levels of specificity and accuracy. Tier 1 relies on globally averaged default values, whereas Tier 2 and Tier 3 require country-specific emission/removal factors or advanced modeling approaches (IPCC, 2006, 2019).

Carbon stock estimation in perennial orchard systems has traditionally relied on a combination of tree allometry, destructive biomass sampling, and soil carbon analysis, although recent trends emphasize the integration of non-destructive techniques and Tier 2 IPCC-aligned methods. The estimation of carbon stock changes in perennial orchard systems has historically been grounded in empirical methods, including destructive biomass sampling, allometric modeling based on tree dimensions, and direct assessments of soil organic carbon. However, recent studies emphasize the need for a broader methodological toolkit—particularly as demonstrated in forest and agroforestry systems. For instance, Nandal et al. (2023b) reviewed four primary approaches for carbon stock estimation: allometric models, eddy covariance, remote sensing, and computer-based models. These include widely used tools such as i-Tree Eco (developed by the United States Department of Agriculture (USDA) Forest Service), Forest-PLUS 2.0 (a joint initiative by United States Agency for International Development (USAID) and India’s Ministry of Environment, Forest and Climate Change), and the Tree Carbon Calculator (CTCC) developed by the Centre for Urban Forestry Research (CUFR). The authors highlight that integrating multiple methods not only reduces estimation uncertainty but also enhances applicability across diverse contexts—including agroforestry, urban forestry, and soil–carbon interaction studies.

**Figure 2:**
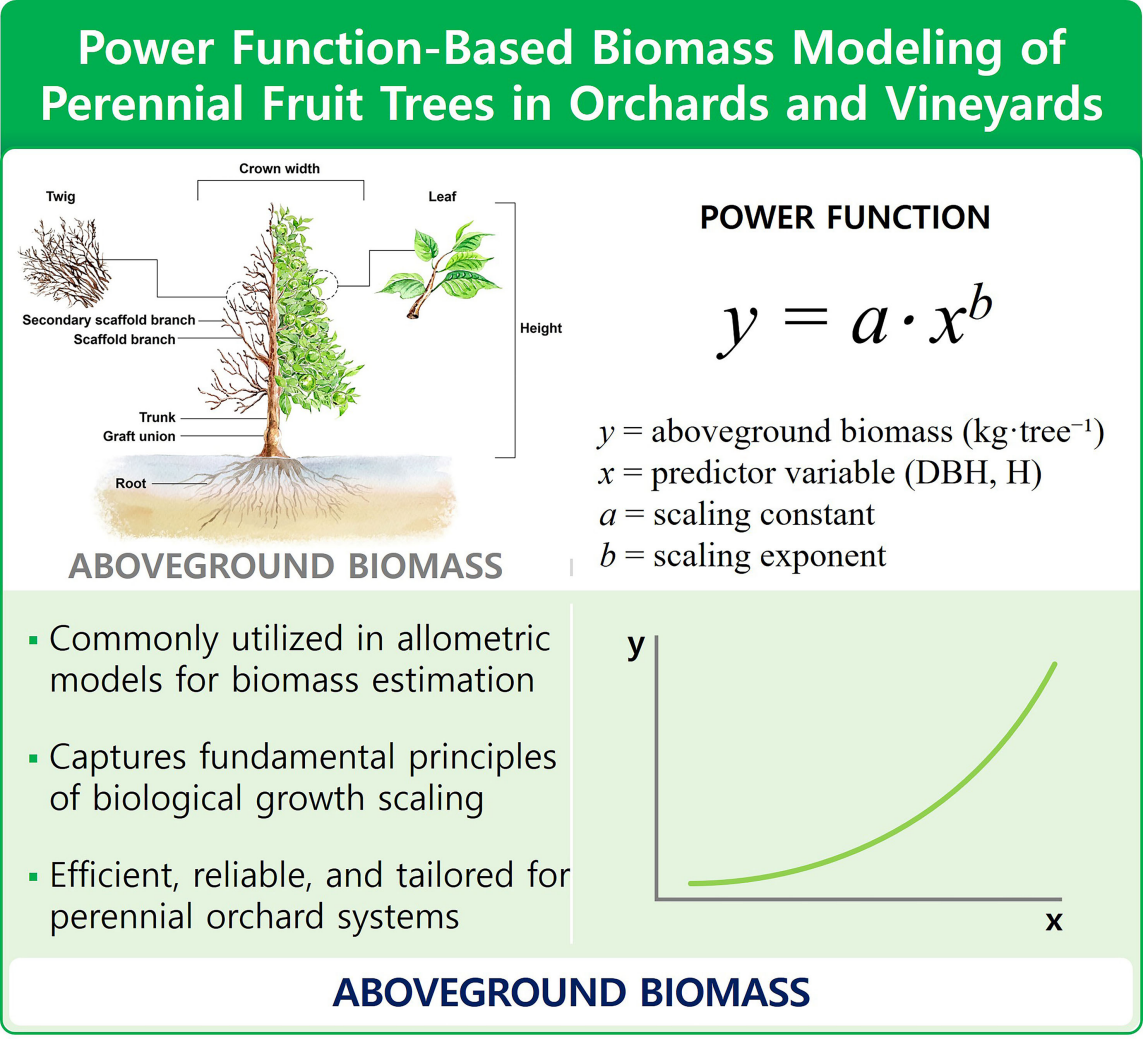
Conceptual framework for applying power functions in biomass estimation of perennial fruit trees in orchards and vineyards. The diagram illustrates how power-law allometric equations (e.g., *y=a·x^b^*) are used to estimate aboveground biomass using biometric variables such as diameter at breast height (DBH) and tree height (H). Power functions are widely adopted in orchard carbon modeling due to their alignment with biological growth scaling, efficiency in field applications, and adaptability to species-specific orchard architectures.

Among the tools used in Tier 2 estimation, allometric equations are particularly essential. These equations define relationships between readily measurable biometric variables—such as diameter at breast height (DBH), tree height, and canopy volume—and aboveground biomass, thereby enabling non-destructive estimation of carbon stocks. This approach is widely regarded as one of the most reliable methods for quantifying carbon at both the individual tree and site scales (Ioannidou et al., 2025; Walker et al., 2016). **Figure 2** shows how tree diameter and height change across developmental stages, and how these biometric attributes are used in allometric modeling to estimate biomass and carbon stocks. The bottom panels depict the application of power function–based regression models that relate stem diameter to aboveground biomass accumulation. This visual integration helps reinforce the empirical basis of carbon estimation methods under Tier 2 and Tier 3 approaches.

Furthermore, selecting models based on plot-level predictive performance, rather than tree-specific performance, minimizes bias and uncertainty when scaling up to hectare-level biomass estimation (Picard et al., 2025). Furthermore, remote sensing approaches—especially airborne Light Detection And Ranging (LiDAR) and high-resolution satellite imagery such as QuickBird—substantially augment the applicability of allometric models by providing precise estimates of canopy height, crown volume, and structural metrics that inform biomass estimation at expanded spatial scales (Borsah et al., 2023). Integrative reviews further highlight how multi-source remote sensing data improves the accuracy and resolution of carbon stock assessments at plot and landscape levels. At the highest precision level, Tier 3 methodologies integrate country-specific allometric models with temporally disaggregated remote sensing and model-based inventory systems, enabling dynamic and location-specific biomass estimation tailored to national circumstances and management practices (IPCC, 2019).

## 3 Results

### 3.1 Overview of selected literature

A total of 240 records were initially retrieved from five electronic databases—Google Scholar, Web of Science, Scopus, Wiley Online Library, and ScienceDirect—using predefined search strings (**Figure 1**). After removing 42 duplicates, 198 unique studies were screened for relevance. During the initial screening phase, 100 records were excluded due to insufficient methodological detail or irrelevance to perennial fruit-tree systems. Full texts were retrieved for the remaining 98 studies, all of which were successfully assessed for eligibility.

After applying the inclusion and exclusion criteria, 53 studies were retained. These included peer-reviewed journal articles, conference papers, and technical reports that developed or applied allometric biomass models for perennial fruit orchards (**Table 1**). The screening and selection process is illustrated in **Figure 1**, following the PRISMA (Preferred Reporting Items for Systematic Reviews and Meta-Analyses) 2020 guidelines.

Studies that focused exclusively on forest trees, shrub species, or annual crops; that lacked biomass quantification or allometric model development; or that reported biomass stocks without equation-based estimation or methodological transparency were explicitly excluded.

Although the review prioritized peer−reviewed articles, SCI−indexed literature specifically addressing orchard-based allometric modeling was found to be limited. This highlights a broader research gap: while forest biomass modeling has advanced significantly across temperate and tropical regions, fruit tree–based models remain underrepresented, particularly within the context of national GHG inventories. The 53 selected studies (summarized in **Table 2**) span a wide range of species, modeling purposes, and geographic regions, reflecting both regional needs and persistent global gaps in orchard carbon accounting (**Table 3**).

**Table 2:** Categorized literature survey on allometric modeling and carbon sequestration in fruit orchards.

Category	Description	Number of studies
Orchard-Based Carbon and Biomass Modeling Studies	Modeling biomass using variables such as age, DBH, tree height, crown width, collar diameter, trunk base diameter, etc.	[51] Aguaron and Roberts (2013); Anthony (2013); Arkalgud et al. (2023); Boongla et al. (2025); Brunori et al. (2017); Bwalya (2013); Callesen et al. (2023); Canaveira et al. (2018); Dao et al. (2021); Darul’aludin et al. (2024); Ganeshamurthy et al. (2016); Génard et al. (2008); Ioannidou et al. (2025); Ita (2020); Kuyah et al. (2024); Ledo et al. (2018); Liguori et al. (2009); Livingston and Lincoln (2023); Mehta et al. (2016); Miranda et al. (2017); Morande (2015); Naik et al. (2018); Naik et al. (2019a); Naik et al. (2019b); Naik et al. (2021); Nemeth et al. (2017); Nogueira et al. (2025); Oumasst et al. (2024); Pacchiarelli et al. (2022); Panumonwatee et al. (2025); Peßler (2012); Plénet et al. (2022); Quiñones et al. (2013); Rathore et al. (2018); Rupa et al. (2020); Sahoo et al. (2021); Salunkhe et al. (2021); Sasaki et al. (2021); Scandellari et al. (2016); Schindler et al. (2023); Song et al. (2023); Sorgonà et al. (2018); Velázquez-Martí et al. (2014); Vercambre et al. (2024); Wu et al. (2012); Yasin et al. (2021); Zanotelli (2012); Zanotelli et al. (2013); Zanotelli et al. (2015); Zanotelli et al. (2018); Zhang et al. (2021)
Allometric models in fruit orchards	Creation of species-specific equations using biometric variables to predict biomass	[23] Arkalgud et al. (2023); Brunori et al. (2017); Canaveira et al. (2018); Dao et al. (2021); Darul’aludin et al. (2024); Ganeshamurthy et al. (2016); Ioannidou et al. (2025); Kuyah et al. (2024); Ledo et al. (2018); Liguori et al. (2009); Livingston and Lincoln (2023); Mehta et al. (2016); Miranda et al. (2017); Naik et al. (2019a); Panumonwatee et al. (2025); Rathore et al. (2018); Rupa et al. (2020); Sahoo et al. (2021); Song et al. (2023); Wu et al. (2012); Zanotelli (2012); Zanotelli et al. (2013); Zhang et al. (2021)
Carbon sequestration in orchards	Assessment of fruit orchards’ roles in GHG inventories and mitigation	[53] Adak et al. (2018); Akram et al. (2017); Anthony (2013); Arkalgud et al. (2023); Boongla et al. (2025); Bwalya (2013); Callesen et al. (2023); Dao et al. (2021); Darul’aludin et al. (2024); Ganeshamurthy et al. (2016); Génard et al. (2008); Granata et al. (2020); Ioannidou et al. (2025); Ita (2020); Kuyah et al. (2024); Kwak et al. (2023); Liguori et al. (2009); Livingston and Lincoln (2023); Mehta et al. (2016); Morande (2015); Murphy et al. (2013); Muthuri et al. (2024); Naik et al. (2018); Naik et al. (2019a); Naik et al. (2019b); Naik et al. (2021); Nemeth et al. (2017); Nimbalkar et al. (2017); Nogueira et al. (2025); Pacchiarelli et al. (2022); Panumonwatee et al. (2025); Peßler (2012); Plénet et al. (2022); Quiñones et al. (2013); Rathore et al. (2018); Rupa et al. (2020); Rupa et al. (2022); Sahoo et al. (2021); Salunkhe et al. (2021); Sasaki et al. (2021); Scandellari et al. (2014); Scandellari et al. (2016); Schindler et al. (2023); Song et al. (2023); Sorgonà et al. (2018); Vercambre et al. (2024); Wu et al. (2012); Yasin et al. (2021); Zanotelli (2012); Zanotelli et al. (2013); Zanotelli et al. (2015); Zanotelli et al. (2018); Zhang et al. (2021)

Summary of 53 peer-reviewed studies, categorized according to their primary research focus. These studies are grouped into three categories: (i) [51 studies] orchard-based carbon and biomass modeling using biometric variables (e.g., age, DBH, tree height, crown width, collar diameter, trunk base diameter); (ii) [23 studies] development of species-specific allometric equations in fruit orchards; (iii) [53 studies] evaluation of carbon sequestration and greenhouse gas (GHG) mitigation potential in orchard systems. Some studies are classified into more than one category; therefore, the category totals exceed the number of unique studies.

**Table 3:** Case studies on carbon sequestration estimates using allometric biomass models in perennial orchard systems.

Orchard type	Reference	Estimated carbon sequestration	Key model variables	Key insights
Citrus	Quiñones et al. (2013)	11.4 Mg C ha^−1^ yr^−1^ (mature trees)	Canopy volume, LAI	Non-destructive models enable broader application across landscapes
Mango	Rupa et al. (2020)	76.2–78.5 t C ha^−1^	Component-specific destructive sampling, growth adjustment	Species-specific models critical for accuracy; varietal diversity less influential
Argan	Oumasst et al. (2024)	0.20 t CO_2_ ha^−1^ yr^−1^ (2- to 6-year-old)	Diameter, height, age	First allometric model for orchard-grown Argan; foundational for expansion
Vineyard	Song et al. (2023)	50.22–61.06 t C ha^−1^ (5- to 20-year-old)	Stem diameter	Carbon storage increases with vine age; Perennial organs hold most biomass carbon

Carbon values are presented as total or annualized carbon sequestration depending on the study. AGB, aboveground biomass; LAI, leaf area index. All cited studies applied allometric relationships with varying levels of validation and uncertainty analysis, emphasizing the importance of species-specific modeling for Tier 2 reporting under IPCC guidance. This table presents selected case studies demonstrating the application of allometric models for estimating aboveground biomass and carbon sequestration across representative perennial orchard types, including citrus, mango, argan, and vineyards. Each example highlights the modeling variables used, estimated carbon stocks, and key methodological insights. These cases underscore the need for species-specific modeling to improve the accuracy and transparency of Tier 2 GHG reporting under IPCC guidelines.

### 3.2 Study distribution and characteristics

Among the 53 peer−reviewed studies analyzed, the majority originated from Asia—particularly India, China, and Thailand—followed by Europe (notably Italy, Spain, and France) and Africa (primarily Kenya and Burkina Faso). This geographic pattern reflects a strong regional concentration of orchard biomass research in Asia and the Mediterranean zone, with limited representation from other tropical regions. The dominant perennial fruit species investigated were mango (n = 5), citrus (n = 5), apple (n = 3), grape (n = 4), olive (n = 2), guava (n = 2), and single-study cases of pomegranate (n = 1), durian (n = 1), breadfruit (n = 1), avocado (n = 1), and longan (n = 1). These studies applied a variety of allometric functions—including power-law, logarithmic, polynomial, and logistic models—reflecting methodological diversity across different climatic regions and orchard systems (**Table 4**).

**Table 4:** Summary of allometric equations for biomass estimation in perennial fruit trees.

Plant category	Scientific name	Allometric fit model	R²	Source
Pomegranate	*Punica granatum*	y=0.215x^1.998^	0.9790	Arkalgud et al. (2023)
Longan	*Dimocarpus longan*	y=1.1116(x^2^H)^0.6537^	0.7825	Brunori et al. (2017)
Olive	*Olea europaea*	y=0.1202x^2.2159^	0.9960	Brunori et al. (2017)
Grape	*Vitis vinifera*	y=4.01x^0.697^	0.7960	Miranda et al. (2017)
Olive	*Olea europaea*	$\text{y}=\frac{19.454}{1+e^{-1.886(a-5.143)}}$	0.5860	Canaveira et al. (2018)
Grape	*Vitis vinifera*	$y=\frac{11.683}{1+e^{-2.232(a-10.439)}}$	0.410	Canaveira et al. (2018)
Fruit trees	*110 data entries*	$y=\frac{18.590}{1+e^{-2.906(a-4.968)}}$	0.2320	Canaveira et al. (2018)
Mango	*Mangifera indica*	y=−2.6554+2.2630ln(D)	0.9546	Dao et al. (2021) Panumonwatee et al. (2025)
Mango	*Mangifera indica*	y=0.083×μDPB^2.184^	0.93	Kuyah et al. (2024)
Avocado	*Persea americana*	y=0.0638×μDPB^2.5435^	0.86	Kuyah et al. (2024)
Durian	*Durio zibethinus*	y=exp[−2.134+2.53ln(D)]	0.86	Darul’aludin et al. (2024)
Mango	*Mangifera indica*	y=2.886(PBG×NPB)^1.039^	0.971	Ganeshamurthy et al. (2016) Rupa et al. (2020)
Mango	*Mangifera indica*	y=2.93(DBGU)^1.22^	0.902	Ganeshamurthy et al. (2016)
Apple	*Malus domestica*	y=0.683(AGE)x^1.76^		Ioannidou et al. (2025) Ledo et al. (2018)
Citrus		y=0.395(AGE)x^2.12^		Ledo et al. (2018)
Orange	*Citrus sinensis*	*y*=1.2069x–19.201	0.9995	Liguori et al. (2009)
Breadfruit	*Artocarpus altilis*	y=−4.586+0.1635x+0.2229x^2^	0.98	Livingston and Lincoln (2023)
Citrus	*Citrus reticulata*	y=2.534+1.013(D)	0.986	Mehta et al. (2016)
Citrus	*Citrus reticulata*	y=7.011+0.019(D^2^H)	0.902	Mehta et al. (2016)
Citrus	*Citrus reticulata*	y=−3.628+6.956ln(D)	*0.982*	Mehta et al. (2016)
Citrus	*Citrus reticulata*	y=−3.016+2.705ln(D^2^H)	0.992	Mehta et al. (2016)
Mango	*Mangifera indica*	y=1.77x^1.02^	0.932	Naik et al. (2019a)
Guava	*Psidium guajava*	y=3.264x^1.012^	0.928	Naik et al. (2021)
Guava	*Psidium guajava*	y=0.0914x^2.4507^	0.998	Rathore et al. (2018)
Orange	*Citrus sinensis*	y=(exp(0.79+0.20(lnD^2^H)))×1.08	0.74	Sahoo et al. (2021)
Apple	*Malus domestica*	y=202.9x^1.61^	0.9105	Zanotelli (2012) Zanotelli et al. (2013)
Grape	*Vitis vinifera*	y=0.8649x^1.1208^	0.9150	Zhang et al. (2021)
Apple	*Malus domestica*	y=0.124x^1.234^	0.984	Wu et al. (2012)
Grape	*Vitis vinifera*	y=0.0223x^2.2023^	0.9695	Song et al. (2023)

Where appropriate, mathematical transformations such as natural logarithmic (ln), square (x²), and exponential (exp) functions were applied to improve model fit and linearize the relationship between biomass and biometric variables. In the listed allometric equations, *y* denotes the estimated aboveground biomass (kg·tree^−1^ or kg·plant^−1^). *x* represents the independent predictor variable, which varies depending on species characteristics and model structure. Commonly used variables include: *a*: crop age; *D*: Diameter at breast height (DBH), measured at 1.3 m above the ground (cm); *H*: Total tree height (m); *AGE*: Age of the tree or age of the aboveground portion (years); *DPB*: Mean diameter at pruned branch base or main stem (cm), often used in orchard trees with regular pruning; *DBGU*: Diameter below graft union (cm), particularly applicable to grafted fruit trees like mango; *PBG*: Primary branch girth (cm), i.e., the basal circumference of main structural branches; *NPB*: Number of primary branches supporting the crown (count); Some equations employ natural logarithmic (ln) or exponential (exp) functions, or compound terms such as D²H to improve model fit and better represent nonlinear growth patterns.

Biometric predictors most frequently used in allometric modeling included DBH, tree height, crown diameter or volume, and basal diameter—corresponding with model inputs listed in **Table 2**. The dominant model form was the power−law function (*y*=*ax^b^*), with mean R² values typically exceeding 0.90 for species−specific models (e.g., Rathore et al., 2018; Ganeshamurthy et al., 2016).

Model performance was assessed using R², the Root Mean Square Error (RMSE), and Akaike Information Criterion (AIC) in most cases, although < 20% of studies incorporated uncertainty quantification or validation across multiple sites (**Table 2**). Notably, studies using integrated approaches—combining destructive and non-destructive measurements or linking allometry with remote sensing—showed improved accuracy (R² > 0.95). Major methodological gaps identified include (i) underrepresentation of belowground biomass and soil−carbon pools, (ii) limited cross−validation across sites or species, and (iii) lack of standard uncertainty reporting consistent with IPCC Tier 2–3 protocols.

### 3.3 Use of power functions in allometric biomass modeling

Allometric equations have long been utilized to estimate biomass in forest and agroforestry systems, including perennial fruit orchards. Among the various functional forms, power-law models have emerged as the most prevalent due to their theoretical grounding in biological growth processes and their empirical reliability (Picard et al., 2012; Poorter and Sack, 2012).

The power function, typically expressed as *y = ax^b^*, where *y* is biomass and *x* is a predictor variable (e.g., stem diameter or tree height), enables researchers to capture nonlinear relationships between tree size and biomass accumulation. This form aligns with the allometric scaling principles that govern plant growth under physical and physiological constraints (Brym and Ernest, 2018; Luo et al., 2019).

As Zanotelli (2012) and Miranda et al. (2017) demonstrated, power functions are especially effective in modeling the biomass of vegetative structures in orchard species. The coefficients *a* and *b* represent biologically meaningful parameters—namely, the scaling constant and the scaling exponent—and their estimation provides insights into species-specific growth behavior.

Panumonwatee et al. (2025) emphasized that while alternative regression forms (e.g., linear, exponential, logistic) may be used, the power function remains the default due to its simplicity and robustness, especially in cases where destructive sampling is impractical.

Similarly, Dao et al. (2021) reported that although both linear and power models provided a superior fit for mango biomass data, the power model outperformed the others when using variables such as DBH and canopy diameter.

Additional studies, such as those by Ganeshamurthy et al. (2016), have further validated the applicability of the power function in commercial orchards, particularly for grafted fruit trees like mango, where pruning practices significantly influence tree architecture. They compared power, logistic, and Gompertz models and concluded that the power form was more straightforward to apply across field data and remained highly accurate.

In a broader context, power-law–based allometric models have become a cornerstone of national and global carbon stock inventories, including those used in IPCC Tier 2 and Tier 3 reporting. Schindler et al. (2023) note that such models facilitate the quantification of carbon stocks in agroforestry systems, providing essential input for climate mitigation strategies and the valuation of ecosystem services.

Importantly, the power-law form is grounded in relative growth theory, which posits that increases in biomass scale predictably with other physical dimensions such as length, surface area, or volume (Chen and Shiyomi, 2019; Niklas, 1994). These biological underpinnings reinforce the model’s relevance across diverse crop types, planting densities, and environmental conditions, including high-density orchards and vineyards.

Ultimately, power-law models in biomass estimation are a product of their biological realism, statistical performance, and ease of use in field-based measurements (Brym and Ernest, 2018; Luo et al., 2019). As such, they are indispensable in orchard carbon accounting and provide a scalable tool for developing Tier 2 and Tier 3 GHG inventories for the AFOLU sector.

### 3.4 Literature trends in allometric modeling and carbon sequestration in fruit orchards

From the final pool of 53 studies, 51 were identified as employing biometric variables to develop species-specific or generalized allometric biomass equations (**Table 2**). These studies varied in species composition, geographic coverage, and modeling approaches.

Accurate estimation of carbon sources and sinks is essential for effective climate-change mitigation, particularly within the AFOLU sector, which accounts for approximately 20–24% of global greenhouse gas emissions (IPCC, 2019). Perennial fruit orchards, as long-lived agroecosystems, have substantial potential to sequester carbon through long-term biomass accumulation. However, reliable quantification of this sink capacity requires robust methods for estimating tree biomass and carbon content—tasks typically achieved through species-specific allometric equations.

Allometric models describe empirical relationships between easily measurable tree attributes such as trunk diameter, height, and canopy volume and total biomass. These models enable non-destructive estimation of carbon stocks and have become essential tools in both ecological research and national carbon accounting. While such models are well established in forestry, comparatively fewer studies have addressed fruit-orchard systems, despite their growing importance for Tier 2 GHG inventory reporting (Lee and Lee, 2022).

**Table 2** provides an overview of peer-reviewed studies that developed or applied allometric models for perennial fruit-tree systems. These studies employed biometric variables such as diameter at breast height (DBH), tree height, crown width, collar diameter, and trunk base diameter to estimate aboveground biomass across diverse orchard species—including mango, citrus, grape, olive, and apple—using both destructive and non-destructive methods.

Out of these, 23 studies specifically focused on the development of species-specific allometric equations, highlighting a growing effort to improve model precision by accounting for species-specific architecture, management practices, and growing environments (e.g., Arkalgud et al., 2023; Dao et al., 2021; Rathore et al., 2018). These models frequently adopt power functions or other nonlinear regression forms, which have proven effective in capturing the growth dynamics of fruit-bearing trees.

Additionally, 53 studies explored the potential of orchards in carbon sequestration, either by directly quantifying biomass or by integrating biomass estimates into national GHG inventories (e.g., Ledo et al., 2018; Panumonwatee et al., 2025; Schindler et al., 2023).

This illustrates the growing recognition of perennial orchard systems as critical components in climate change mitigation strategies, particularly within the IPCC’s AFOLU sector framework.

Taken together, the body of literature summarized in **Table 2** highlights not only the methodological diversity of biomass modeling approaches but also the growing scientific and policy interest in integrating orchard systems into national carbon accounting frameworks and ecosystem service valuation strategies.

**Table 3** presents a comparative summary of recent case studies that have developed or applied allometric biomass models to estimate carbon sequestration in perennial orchard systems. These studies span a wide range of climates, species, and management practices—including citrus, mango, argan, and grapevine systems—demonstrating both the methodological diversity and ongoing progress in integrating orchard systems into national carbon accounting frameworks (Oumasst et al., 2024; Quiñones et al., 2013; Rupa et al., 2020; Zhang et al., 2021; Song et al., 2023).

Across these examples, allometric models have been tailored to species-specific morphological traits and orchard management regimes. Methodological approaches vary from non-destructive canopy-based estimation to component-wise destructive sampling, depending on the research objective and developmental stage of the trees. For instance, Quiñones et al. (2013) employed canopy volume and LAI in mature citrus trees, while Oumasst et al. (2024) developed allometric functions for young cultivated argan trees based on age, height, and diameter. Rupa et al. (2020) used destructive sampling to differentiate biomass allocation in mono- and multi-varietal mango systems, whereas Zhang et al. (2021) and Song et al. (2023) demonstrated the long-term carbon storage potential of vineyards, with carbon accumulation increasing with vine age.

These case studies collectively underscore the importance of context-specific modeling to improve the accuracy of biomass and carbon estimates in orchard ecosystems. Such models are increasingly essential for supporting Tier 2 reporting under the IPCC guidelines, where species- and management-specific emission/removal factors are required to enhance transparency, consistency, and accuracy in GHG inventories (IPCC, 2006, 2019; Smith et al., 2014).

### 3.5 Overview of allometric equations for biomass estimation in perennial fruit trees

**Table 4** summarizes a diverse set of published allometric equations that apply a range of functional forms—including power functions, logarithmic models, polynomial regressions, and sigmoid growth curves—for estimating aboveground biomass and carbon stocks in perennial fruit orchards. While power-law models such as y=ax^b^ remain dominant due to their biological grounding (e.g., y=0.215x^1.998^, y=0.124x^1.234^), other forms including log-transformed (y=−2.6554+2.2630lnD), polynomial ((y=−4.586+0.1635x+0.2229x²), and logistic sigmoid functions (y =19.454/[1+e^(1.886(x-5.143))^]) are also widely used depending on species, regional context, and measurement variables such as diameter, tree height, age, or crown dimensions. These equations span diverse species, including mango (*Mangifera indica*), citrus (*Citrus* spp.), olive (*Olea europaea*), apple (*Malus domestica*), grape (*Vitis vinifera*), and others. The diversity of functional forms and biometric variables used reflects species-specific growth characteristics, pruning practices, and the need for tailored carbon accounting in orchard systems.

In most equations, the dependent variable (y) represents the aboveground biomass (typically in kg·tree^−1^ or kg·plant^−1^), and the independent variables (x) include biometric traits such as diameter at breast height (D, cm), total tree height (H, m), tree age (AGE, years), diameter below graft union (DBGU, cm), and mean diameter at pruned branch base (DPB, cm). Additional variables such as primary branch girth (PBG) and number of primary branches (NPB) are especially relevant in orchard trees with complex crown architecture or intensive management (e.g., Ganeshamurthy et al., 2016; Kuyah et al., 2024).

**Figure 3:**
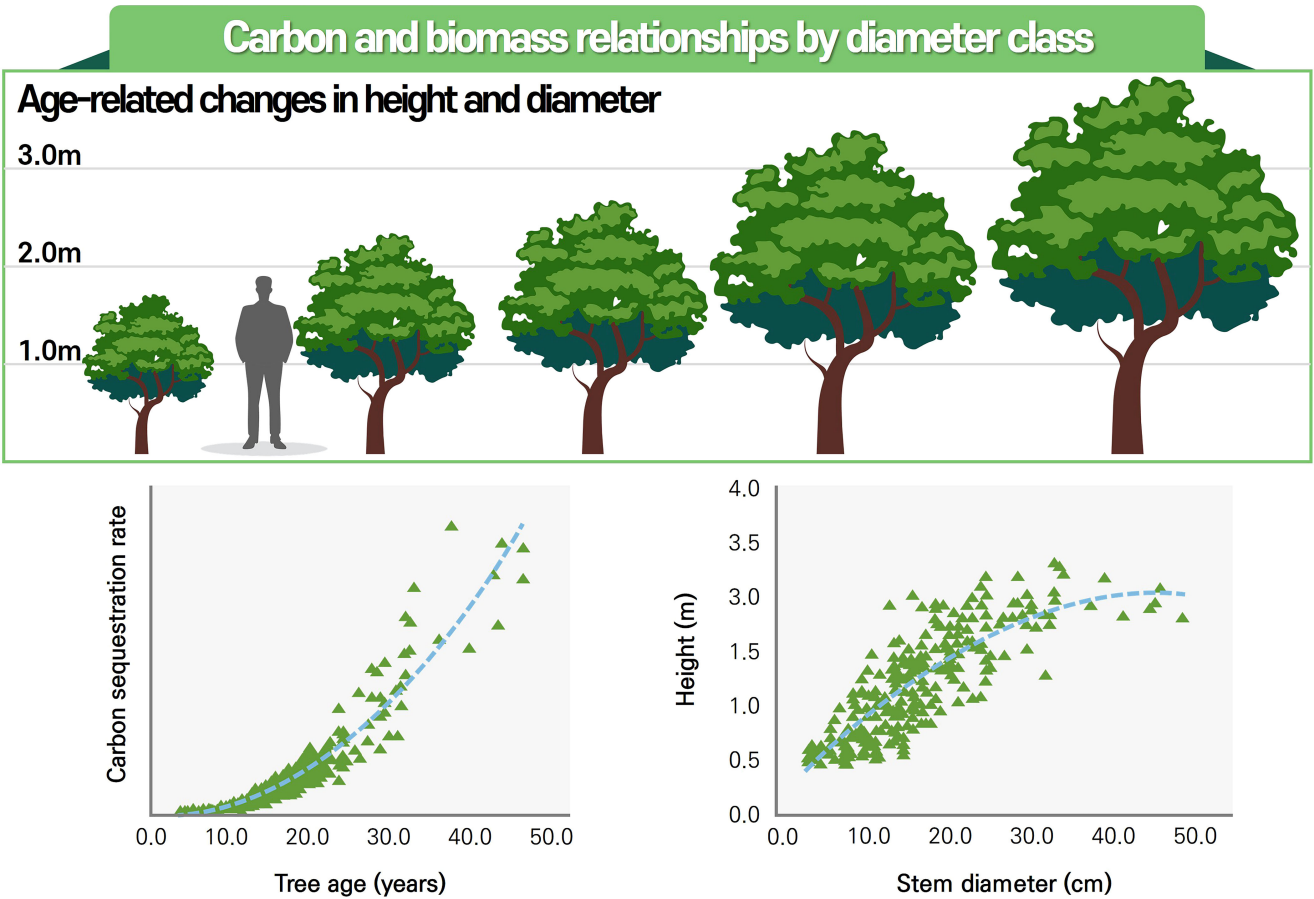
Relationships between tree age, stem diameter, and carbon dynamics. This figure illustrates the relationships among tree age, height, and diameter development (top), along with the corresponding increases in aboveground biomass and carbon stock (bottom left and right), as modeled using allometric power functions. The regression plots highlight nonlinear growth trajectories, showing how biomass accumulation scales with stem diameter across various developmental stages in orchard systems.

Several models adopt power-law or exponential forms to capture the nonlinear relationship between biomass and tree dimensions. For example, Rathore et al. (2018) reported a highly accurate guava model using a power function of stem diameter with an R² of 0.998, while Mehta et al. (2016) employed compound terms such as D²H and logarithmic transformations to model biomass in *Citrus reticulata*. These functional forms are common in allometric modeling as they help linearize biomass relationships and improve model fit (Ledo et al., 2018; Dao et al., 2021). **Figure 3** illustrates the general structure and conceptual basis of the power function commonly used in allometric biomass models for perennial fruit trees.

Some studies also applied logistic functions to describe biomass accumulation over time, especially for trees such as mango and grapevine where growth tends to saturate with age (Canaveira et al., 2018; Song et al., 2023). These models are particularly useful in perennial systems where tree age significantly influences biomass dynamics.

While species-specific models demonstrated high predictive power (e.g., Liguori et al., 2009 for orange, R² = 0.9995), generalized models based on pooled datasets from multiple fruit tree species yielded lower R² values (e.g., Canaveira et al., 2018; R² = 0.2320), emphasizing the importance of targeted model development.

Collectively, this synthesis illustrates the importance of allometric equations in enabling accurate and scalable biomass estimation for orchard-based systems. The inclusion of these equations in national carbon accounting frameworks may contribute to more refined Tier 2 and Tier 3 reporting for the AFOLU sector under the IPCC guidelines.

## 4 Discussion

### 4.1 Advancing carbon accounting in perennial orchard systems: toward a standardized methodological framework

The global intensification of climate change has underscored the importance of robust and transparent national GHG inventories. The 2015 Paris Agreement requires all signatory countries to submit updated Nationally Determined Contributions (NDCs) every five years and to publish annual National Inventory Reports (NIRs) to track their emissions and progress toward mitigation targets (UNFCCC, 2015).

**Figure 4:**
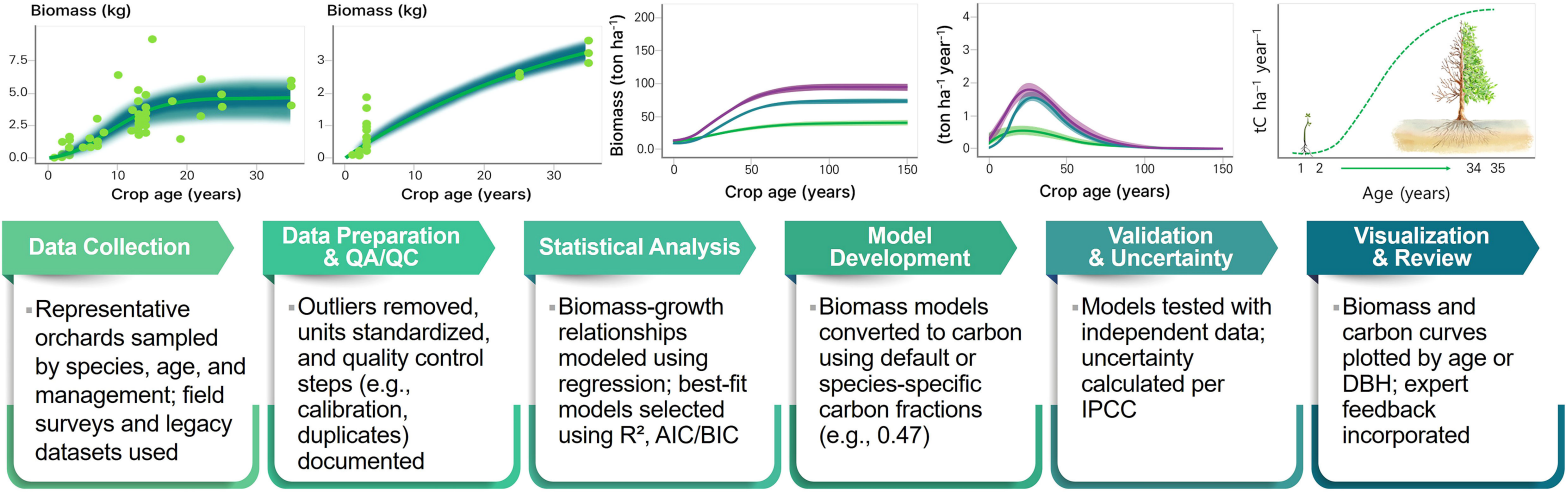
Methodological frameworks for developing biomass-based carbon emission and removal factors for perennial orchard systems in accordance with IPCC guidelines. The six-step process includes data collection, quality assurance and quality control (QA/QC), statistical analysis, model development, validation and uncertainty assessment, and final visualization with expert review. This approach ensures transparency, accuracy, and representativeness for national greenhouse gas inventories.

As emphasized throughout this review, the development and application of species-specific allometric equations are essential for improving the accuracy of biomass and carbon stock estimations in perennial orchard systems. To operationalize this improvement, **Figure 4** introduces a structured methodological framework designed to support the development of orchard-specific carbon emission and removal factors in accordance with IPCC Tier 2 and Tier 3 guidance (IPCC, 2006, 2019). Anchored in the IPCC’s core principles of transparency, consistency, comparability, completeness, and accuracy (TCCCA), this framework outlines a robust and replicable process from empirical data collection through model development to inventory integration.

In particular, long-lived perennial orchard systems, such as apple or persimmon orchards, act as stable carbon sinks over multiple decades due to sustained structural biomass and root turnover. Their sequestration potential can be further enhanced by climate-smart practices including pruning residue retention, inter-row grass cover, and optimized planting density. However, these contributions remain insufficiently quantified in national GHG inventories, largely due to the lack of species-specific and age-stratified data.

While mature orchard trees dominate current datasets, juvenile trees often exhibit disproportionately higher root-to-shoot ratios and distinct allocation patterns, particularly in early establishment phases. Neglecting these dynamics may lead to systematic bias in estimating carbon accumulation trajectories across orchard lifecycles.

Empirical data on belowground biomass (BGB) remain extremely limited, primarily due to the destructive and labor-intensive nature of root excavation. As a result, even well-established orchard systems often lack direct root measurements, which introduces significant uncertainty into biomass estimates during early and mature growth phases. This lack of empirical data hinders progress toward Tier 3 approaches, especially in systems where root biomass plays a central role in long-term carbon sequestration.

Geographically, the reviewed datasets are heavily concentrated in East Asia (e.g., Korea, Japan, and China) and Southern Europe (e.g., Italy and Spain), with notable gaps in data from sub-Saharan Africa, Central Asia, and Latin America. Moreover, orchard form and training styles—such as bush-type, espalier, or multi-leader systems—vary by country, further complicating model applicability across different agroecological settings.

Furthermore, grafted orchard trees—which dominate most commercial fruit production—introduce structural discontinuities between rootstock and scion, affecting wood density, hydraulic conductance, and biomass partitioning. Despite their prevalence, few allometric models explicitly account for such graft-induced variations, potentially leading to systematic under- or over-estimation of carbon stocks. As countries increasingly adopt enhanced inventory approaches under Tier 2 and Tier 3 methodologies, the development of robust, empirically grounded protocols for orchard-based carbon accounting has become a pressing need (Smith et al., 2014; Henry et al., 2011).

The proposed methodology initiates comprehensive field measurements at representative orchard sites, capturing biometric attributes such as DBH, tree height, and canopy volume, along with detailed site metadata (e.g., soil type, climate zone, management practices, and tree age class) (Henry et al., 2011; Schindler et al., 2023). For example, Schindler et al. (2023) surveyed 70 wild cherry (*Prunus avium*) trees in southwestern Germany using terrestrial laser scanning and quantitative structure models—meticulously measuring DBH, tree height, and canopy volume, while also documenting management regimes and tree age classes—to develop allometric models with high predictive precision (adjusted R² ≥ 0.97). Collected datasets undergo rigorous quality assurance/quality control (QA/QC) procedures, including unit harmonization, outlier detection, and metadata validation, as outlined by the IPCC (2006).

A suite of statistical modeling techniques—both linear and nonlinear—is then applied to develop allometric equations tailored to species and site conditions. Models are selected based on their empirical performance, as evaluated by R², AIC, and Bayesian Information Criterion (BIC) (Picard et al., 2012). The predicted biomass is converted into carbon stock estimates using species-specific or default carbon fractions (commonly 0.47 for woody biomass) in accordance with IPCC defaults (IPCC, 2006).

Model validation follows, using independent datasets to test prediction accuracy with statistical indicators such as RMSE and Mean Error (ME). In parallel, uncertainty quantification is conducted using error propagation techniques recommended in IPCC guidance, enabling the derivation of 95% confidence intervals and combined uncertainty estimates (IPCC, 2019). These steps are critical to enhancing credibility and comparability of the derived emission/removal factors for national reporting.

To support transparency and stakeholder engagement, model outputs are visualized through interpretable tools such as biomass–age curves, DBH–carbon relationships, and site-specific response functions. These visualizations facilitate peer review, decision support, and scenario refinement under national inventory frameworks.

Ultimately, the framework culminates in the derivation of Tier 2-specific emission/removal factors, fully documented with spatial and temporal boundaries, methodological justifications, and associated uncertainty ranges. This standardized yet flexible protocol offers a science-based pathway to formally integrate perennial orchard systems into national AFOLU inventories, advancing GHG mitigation strategies in alignment with international climate commitments.

### 4.2 Integrating perennial orchards into IPCC reporting and national inventory systems

The framework presented in this review has important implications for countries seeking to improve the accuracy, transparency, and representativeness of their GHG inventories under the LULUCF sector. In particular, the inclusion of perennial orchard systems within Tier 2 and Tier 3 reporting frameworks addresses a long-standing omission of perennial agricultural systems from national carbon accounting.

Several countries, such as Switzerland, Australia, and the Netherlands, have begun to incorporate differentiated emission factors for orchard systems based on species-specific allometric equations and remote sensing–based stratification (UNFCCC, 2024). However, many national inventories continue to rely on Tier 1 default values, which do not capture the variability introduced by species traits, pruning intensity, planting density, and management regimes.

Notably, vineyard systems present unique challenges for allometric modeling due to their multi−stemmed architecture, renewal pruning cycles, and high inter−annual variability. Despite their wide geographical distribution and long−term presence in Mediterranean and temperate zones, vineyards remain underrepresented in AFOLU inventories, highlighting the need for tailored modeling frameworks that reflect their structural and physiological distinctiveness.

Furthermore, a persistent bias exists in the dominant use of power-law and log-linear allometric models—these forms are favored for their statistical robustness, ease of linearization, and relatively good fit with limited datasets. Yet, such choices often prioritize empirical stability over biological realism, potentially obscuring nonlinear growth trajectories or physiological thresholds inherent in tree development.

The implications of grafted tree structures, commonly found in orchard systems, further complicate model development. Rootstock–scion interactions may alter resource allocation patterns, hydraulic conductance, and wood density, introducing additional uncertainty into biomass predictions. However, few existing models explicitly account for graft-induced structural discontinuities, leading to a potential underestimation or misrepresentation of true carbon stocks.

Age structure is another under-addressed factor in allometric modeling. A substantial number of existing studies focus on mature orchard trees (typically older than 7 years), while data on juvenile or early-stage trees remain scarce. This limits the predictive applicability of models to younger orchards or newly established systems, where growth dynamics differ significantly.

Although perennial orchards are increasingly recognized as important carbon sinks, significant data gaps persist regarding tree form, root biomass, and age-specific allocation, particularly when considering geographic and species-specific variability.

Tree architecture and orchard management practices vary widely across countries and regions due to differing cultivars, pruning styles, planting densities, and training systems. For instance, espalier-trained apple trees in Europe present markedly different biomass allocation patterns than free-standing persimmon trees in East Asia. This spatial heterogeneity is rarely captured in current carbon estimation protocols, introducing regional uncertainty in inventory extrapolations.

In addition, root biomass (i.e., BGB) remains among the least characterized carbon pools. Root-to-shoot ratios—often used as proxy estimators for belowground biomass—fluctuate considerably depending on growth stage, species physiology, and seasonal phenology, especially in deciduous orchards where leaf presence and allocation shift throughout the year. While BGB is a critical contributor to long-term soil carbon storage, its direct measurement is highly impractical due to the labor-intensive, destructive nature of complete root excavation. As a result, country- or forest type-specific allometric equations for roots are scarce, and indirect estimations using aboveground biomass remain the only practical option in many inventory frameworks.

Juvenile orchard trees, though underrepresented in empirical datasets, often display disproportionately higher root-to-shoot ratios and distinct carbon allocation patterns compared to mature trees, especially during early establishment. Overlooking these developmental phases can introduce systematic bias in estimating carbon accumulation across orchard lifespans.

Although the 2006 and 2019 IPCC Guidelines recommend stratifying carbon stock change estimates by age class and rotation cycles, most country-level inventories lack adequate field data for young or newly planted orchards. Such data gaps—if unaddressed—can compromise the accuracy of Tier 2 and Tier 3 carbon accounting, particularly in countries aiming to include perennial orchards in their AFOLU inventories with greater spatial and temporal resolution.

Despite accounting for an estimated 30–40% of ecosystem carbon stocks, root biomass remains underrepresented in species-specific carbon models used in national inventories. This modeling gap constrains the accuracy of Tier 2 and Tier 3 carbon accounting, especially in orchard systems with high site and species variability.

Root-to-shoot ratios also vary by species traits, seasonal phenology, and orchard management stages—especially in deciduous systems. Yet, fewer than 10% of reviewed studies include empirical root data, with most relying on generalized ratios that fail to account for cultivar- or site-specific variability. This represents a major source of error in Tier 2 national inventories.

Geographically, existing datasets are concentrated in East Asia (China, Korea, and Japan) and Southern Europe (Italy and Spain), creating a regional bias that undermines the transferability of derived models to underrepresented zones such as sub-Saharan Africa, South America, and Central Asia. Such gaps pose challenges to global inventory comparability and hinder capacity building in developing regions.

By implementing standardized, empirically derived allometric protocols, countries can significantly reduce estimation bias and uncertainty margins in their national inventory reports. Moreover, this framework aligns with emerging trends in the AFOLU-based mitigation strategies, enabling countries to recognize orchard systems not only as production systems but also as climate mitigation assets. To this end, future research must expand to encompass biologically meaningful model forms, incorporate graft-specific architecture, include juvenile tree stages, and invest in belowground sampling, while actively addressing geographical disparities and methodological blind spots.

Integrating these systems into national inventories would allow policymakers to quantify co-benefits, such as soil carbon enhancement, biodiversity conservation, and sustainable land management. This, in turn, can improve access to climate finance, enhance reporting credibility under the United Nations Framework Convention on Climate Change (UNFCCC), and support nature-based solutions to climate change.

The application of orchard-specific carbon emission factors offers a range of policy-relevant benefits—including improved alignment with the IPCC’s TCCCA principles (transparency, comparability, accuracy, completeness, and consistency), enhanced resolution in the AFOLU subcategory reporting, a solid foundation for incentive mechanisms such as carbon payments and forestry support, and stronger support for achieving national net-zero targets—while the development of Tier 2-specific emission/removal factors enables more accurate and representative greenhouse gas inventories that reflect country-specific ecological and management conditions, thereby serving as a critical foundation for enhancing the credibility of LULUCF sector reporting and its alignment with international reporting frameworks.

While this review advances the methodological framework for orchard−specific carbon accounting, several significant limitations merit discussion. First, review bias may have arisen because our search was restricted to English−language publications and peer−reviewed journals only, which excludes grey literature and non−English research, introducing a language bias and potential under−representation of studies from low−income regions. Second, there are clear geographical data gaps: while most allometric studies have been conducted in Europe, North America, and East Asia, tropical and low-income regions including Africa, South Asia, and Latin America remain critically underrepresented. This imbalance limits the global applicability and transferability of emission and removal factors in carbon accounting frameworks.

Third, belowground biomass pools—including roots, rhizodeposition, and soil organic carbon—are still poorly quantified in orchard systems. These components are often omitted or inferred using forest-based ratios, introducing substantial uncertainties into national inventory estimates.

From a policy perspective, adopting orchard-specific Tier 2 emission/removal factors (Japanese Government, 2024; Australian Government, 2024) enables more accurate and transparent estimation of carbon removals in the cropland-remaining-cropland subcategory, compared to generic Tier 1 defaults (IPCC, 2006).

This shift improves the scientific robustness of national inventories, strengthens the credibility of mitigation reporting, and lays the foundation for carbon crediting and incentive mechanisms in perennial horticulture (IPCC, 2019; UNFCCC, 2024).

In summary, integrating orchard-specific allometric protocols will enhance the accuracy and credibility of national AFOLU inventories.

## 5 Conclusion

Perennial orchard systems constitute an important yet underrepresented component in national strategies for climate change mitigation. This review underscores the need to develop orchard-specific biomass models and emission/removal factors that accurately reflect their carbon sequestration potential. The use of species-specific allometric equations—grounded in empirical field measurements and supported by rigorous validation and uncertainty analysis, enables countries to advance from Tier 1 defaults to Tier 2 reporting under the IPCC framework.

By consolidating existing studies and presenting a standardized methodological framework, this review contributes to the scientific foundation necessary for integrating orchard systems into national GHG inventories. Such integration not only enhances inventory precision and transparency but also facilitates the recognition of fruit orchards as viable nature-based solutions in climate policy. Ultimately, the inclusion of perennial orchard systems in carbon accounting frameworks will help support climate-smart agricultural transitions and strengthen efforts toward achieving national and global carbon neutrality targets.

## Data Availability

The original contributions presented in the study are included in the article/supplementary material, further inquiries can be directed to the corresponding author/s.
